# The effect of Substance P/Heparin conjugated PLCL polymer coating of bioinert ePTFE vascular grafts on the recruitment of both ECs and SMCs for accelerated regeneration

**DOI:** 10.1038/s41598-019-53514-6

**Published:** 2019-11-19

**Authors:** Donghak Kim, Justin J. Chung, Youngmee Jung, Soo Hyun Kim

**Affiliations:** 1 KU-KIST Graduate School of Converging Science and Technology, Korea University, 145 Anam-ro, Seongbuk-gu, Seoul, 02841 Republic of Korea; 2 Center for Biomaterials, Korea Institute of Science and Technology, Seoul, 02792 Republic of Korea; 3 Department of Biomedical Engineering, Korea University of Science and Technology (UST), Daejeon, 305-350 Republic of Korea

**Keywords:** Biomedical materials, Implants

## Abstract

Artificial vascular grafts consisting of ePTFE have been mainly used in clinics for the treatment of cardiovascular disease. However, artificial grafts can become clogged after a long time due to thrombosis, as graft maturation by endothelialization is limited. The strategy introduced in this study is to induce graft remodeling through interaction between the bioinert graft and the body. The Substance P (SP) and heparin were covalently conjugated with PLCL, an elastic biocompatible copolymer and the Substance P-conjugated PLCL (SP-PLCL) and/or heparin-conjugated PLCL (Hep-PLCL) were vacuum-coated onto ePTFE vascular grafts. To assess the effectiveness of the coating, coated samples were evaluated by implanting them subcutaneously into SD-Rats. Coatings allow grafts to be remodeled by creating a microenvironment where cells can grow by infiltrating into the grafts while also greatly enhancing angiogenesis. In particular, a double coating of Hep-PLCL and SP-PLCL (Hep/SP-PLCL) at four weeks showed markedly improved vascular remodeling through the recruitment of mesenchymal stem cells (MSCs), vascular cells (ECs, SMCs) and M2 macrophages. Based on these results, it is expected that when the Hep/SP-PLCL-coated ePTFE vascular grafts are implanted *in situ*, long-term patency will be assured due to the appropriate formation of an endothelial layer and smooth muscle cells in the grafts like native vessels.

## Introduction

**Figure 1:**
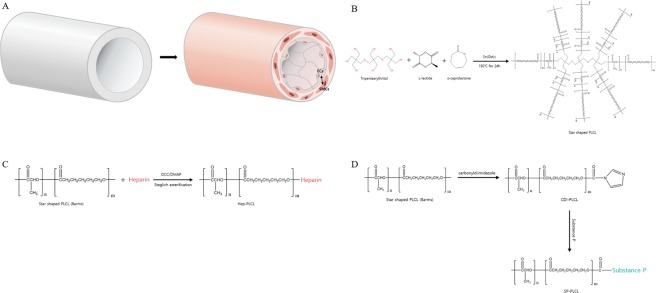
(**A**)Schematic illustration of autologous tissue regeneration on bio-inert teflon vascular graft. Non-coated Teflon vascular graft has no cells but, SP-PLCL and Hep-PLCL coated Teflon vascular graft is remodeled and so has many vascular cells. (**B**) Star shaped PLCL is synthesized by using tripentaerythritol with eight hydroxyl groups as and initiator. (**C**) Conjugation of heparin with star-shaped PLCL using a DCC/DMAP coupling reaction. (**D**) Conjugation of Substance P with star shaped PLCL using 1,1′-carbonuldiimidazole (CDI) chemistry.

Vascular diseases continue to threaten human life. As a clinical treatment method, the transplantation of vascular autografts has served as an effective strategy. However, autologous vessels for autologous bypass grafting cannot always be harvested, and their availability is highly limited at times. Hence, currently artificial vascular grafts are often used. Large-diameter (≥6 mm inner diameter) expanded polytetrafluoroethylene (ePTFE) vascular grafts are mostly used blood vessel applications. However, the issues of poor long-term patency and restenosis can arise after bypass surgery, typically leading to implant failure eventually. Specifically, small-diameter artificial vascular grafts (<6 mm inner diameter) are highly associated with the risk of thrombosis and occlusion and are thus not currently available for clinical use^1,2^. Their inner surfaces are thrombogenic and their intrinsic hydrophobicity can limit endothelium formation^2,3^. Therefore, through a surface coating on ePTFE, the thromboresistance can be increased by preventing nonspecific protein attachment and endothelialization can be induced at the lumen to enhance long-term patency^4–6^. Most research has been focused on adhering and retaining only endothelial cells (ECs) to an ePTFE graft surface through surface modifications such as coating and plasma modification to inhibit protein and platelet adsorption. However, endothelial cells (ECs) attached to ePTFE vascular grafts are properly separated by the blood flow due to the weak interaction with the hydrophobic surface of the ePTFE^7^. However, unlike ECs, smooth muscle cells (SMCs) adhere well to the arterial wall of PTFE and resist blood shear. Moreover, SMC-seeded PTFE has superior EC retention ability as compared to only PTFE^8^. Thus, it is necessary to prevent endothelial cells from being washed out by the blood flow through interaction between smooth muscle cells growing inside ePTFE and endothelial cells of the lumen, similar to a native vessel (Fig. 1(A). In the present study, through a polymer coating, we intended to remodel ePTFE artificial vascular grafts by recruiting SMCs and ECs with bioactive molecules capable of instructing the surrounding tissues to migrate into the graft, thus stimulating the recruitment and differentiation of stem cells, inducing angiogenesis, and promoting regeneration.

Poly(l-lactide-co-ε-caprolactone) (PLCL), a biodegradable and biocompatible polymer, is a very flexible and rubberlike elastic copolymer^9,10^. Accordingly, PLCL has many potential uses in relation to soft tissue regeneration, such as in blood vessels, tendons, skin, esophagus, and cardiac tissues. PLCL is particularly well suited for certain blood vessels because it requires some degree of flexibility to withstand high blood pressures. Thus, we selected PLCL to impart these properties to bio-inert ePTFE vascular grafts and as a carrier for delivery of biomolecules. Also, we used a star-shaped PLCL with eight hydroxyl groups to bond more biomolecules covalently.

Substance P (SP) is a neuropeptide with 11 amino acids which is involved in inflammation, cell proliferation, cell migration and wound healing. SP also has an immunomodulatory effect and induces the production of various growth factors and cytokines. It can induce a regeneration process through the recruitment of endogenous stem cells such as CD29+ stromal cells, MSCs, and HSCs^11–16^, and it induces enhanced angiogenesis through the recruitment of circulating angiogenic cells to injury sites^17^. Finally, SP induces the proliferation and differentiation of endothelial cells and the mitogenesis of smooth muscle cells^12,18^.

Heparin is a highly sulfated glycosaminoglycan that contains sulfonic, sulfoamino, and carboxyl groups. Because heparin has a higher negative charge density than any other biological macromolecule^19^, it prevents thrombus formation by repelling platelets and proteins from surfaces and exerts an anticoagulation effect by binding to antithrombin III^20–23^. Heparin is also a component of the extracellular matrix of blood vessels and promotes endothelial cell growth *in vitro*^24^. It demonstrates numerous important biological activities associated with its interaction with diverse proteins, including enzymes, lipoproteins, viral coat proteins, and extracellular matrix proteins, especially cytokines and growth factors due to the specific electrostatic interactions between the negatively charged sulfate groups of heparin and the positively charged amino acid residues of proteins^20,25–29^.

It is difficult to obtain small-diameter ePTFE vascular grafts because currently they are not available in clinical practice. While large diameter ePTFE grafts are used in clinic, room for performance improvement still exist such as increase of biocompatibility and prevention of thrombosis. Thus, we chose the most widely used 6 mm ePTFE vascular grafts for investigation. And it can be implanted only in large animals such as cows and pigs. Prior to large-animal *in situ* implantation, we needed a definitive verification about the effectiveness of coatings *in vivo* as a precedent research. Therefore, we have previously implanted samples into the subcutaneous tissue and tested the possibility.

In this study, we attempt to remodel bioinert ePTFE artificial vascular grafts using bioactive molecules to resolve the poor long-term patency of ePTFE vascular grafts. To do so, we coated SP conjugated PLCL (SP-PLCL) and Heparin conjugated PLCL (Hep-PLCL) on ePTFE vascular grafts. The angiogenic effect and the recruiting ability of MSCs, surrounding cells and macrophages for regeneration were investigated by subcutaneous implantations into a SD-rat model.

## Materials and Methods

### Synthesis of star-shaped PLCL

Eight armed star-shaped PLCL (50:50) copolymers were synthesized by ring opening polymerization of L-lactide (PURAC biomaterials, seoul, korea) and ε-caprolactone (A10299, Alfa aesar, Massachusetts, United States). Tripentaerythritol (107646, Sigma Aldrich, Missouri, United States) and tin(II) 2-ethylhexanoate (S3252, Sigma Aldrich, Missouri, United States) were used as an initiator and as a catalyst at ratio of 1.25 × ${10}^{-4}$ and 5 × ${10}^{-4}$ moles per mole of total monomers, respectively. L-lactide and ε-caprolactone and initiator and catalyst were put in glass ampoule then, it was purged three times with nitrogen (N2) and vacuum. After vacuum for 6 h, it was heat sealed and transferred to a pre-heated silicon oil bath (150 °C) and then, polymerization was carried out for 24 h. Resultant star-shaped polymer was purified by dissolution in chloroform and filtering with 2.5 μm pore membrane and precipitation with excess methanol. The polymer was washed with new methanol and then was dried 3days in vacuum. The number of average molecular weight (Mn) of star-shaped PLCL was about 80,000 *gmol*^−1^, as measured by gel permeation chromatography.

### Substance P conjugated PLCL fabrication

Substance P (97% purity, Peptron, Daejeon, Korea) was covalently conjugated with star-shaped PLCL (SP-PLCL) using CDI chemistry (Fig. 1(D)). 12 g Star-shaped PLCL was dissolved in 70 ml DCM with stirring for 24 h and 0.5 g CDI (115533, Sigma Aldrich, Missouri, United States) was dissolved in 10 ml DCM with stirring for 12 h. CDI solution was added in PLCL solution and stirred for 24 h under N2. CDI activated PLCL (CDI-PLCL) was precipitated with excess methanol and dried in a vacuum oven for 3 days. 4 g CDI-PLCL was dissolved in 40 ml DCM with stirring for 24 h under N2 and 15 mg SP was dissolved in 20 ml DMSO on vortex for 12 h. SP solution was added in CDI-PLCL solution and then reaction was carried out for 48 h under N2 at RT. SP-PLCL was precipitated with excess methanol and then dried in a vacuum oven for 3 days^14,27^.

### Heparin conjugated PLCL fabrication

Heparin (H3393, Sigma Aldrich, Missouri, United States) was covalently conjugated with star-shaped PLCL (Hep-PLCL) using DCC/DMAP chemistry as described Fig. 1(C). 0.8 g of heparin was dissolved in a mixture of 55 ml formamide and 55 ml N,N-dimethylformamide with stirring for 3 days. 12 g star-shaped PLCL was dissolved in 150 ml methylene chloride with stirring for 24 h. 55 mg DCC (D80002, Sigma Aldrich, Missouri, United States) and 37.5 mg DMAP (107700, Sigma Aldrich, Missouri, United States) were added in heparin solution with stirring for 10 min. The polymer solution was dropped slowly into the heparin solution and stirred for 24 h at 50 °C under N2. After conjugation reaction, reaction system was concentrated and the precipitated with excess ethanol. The precipitate was washed with distilled water and then dissolved in chloroform for purification. The obtained solution was reprecipitated with excess methanol and Hep-PLCL was dried in a vacuum oven for 3 days^14,30^.

### Toluidine blue assay

Heparin content of Hep-PLCL was determined by the toluidine blue colorimetric method. Since the polymer was aggregated as it is precipitated and dried, almost heparin on the surface was quantified. 50 mg of Hep-PLCL was placed in 300 µl of 0.01 M HCL aqueous solution containing 0.2% NaCl, after which 200 µl of 1X PBS and 500 µl of toluidine blue (Alphachem, Mississauga, Canada) solution (8 mg of toluidine blue dissolved in 20 ml of 0.01 M HCL aqueous solution containing 0.2% NaCl) was added. After vortexing for 4 h, sample was rinsed with 1X PBS 3 times for removing free toluidine blue. And then 200 µl of EtOH solution containing 0.1 M NaOH (mixture of 160 µl EtOH and 40 µl of 0.1 M NaOH) are added. Then absorbance of resulting solution at 620 nm was determined by a UV spectrophotometer. The heparin content on the surface of Hep-PLCL can be quantified through standard curve of absorbance at 620 nm with different concentration of heparin prepared by the same method. Standard curve at 0.2–100 µg/ml heparin concentrations was established to fit a linear relationship in the range^31–33^.

### X-ray photoelectron spectroscopy (XPS)

Elemental analysis using X-ray photoelectron spectroscopy (XPS) was performed to verify the incorporation of substance P by confirming the presence of nitrogen peak. XPS spectra of the powders were obtained using PHI 5000 VersaProbe (Ulvac-PHI) with Al Kα radiation (1486.6 eV).

### Fourier transform infrared spectroscopy (FTIR)

Attenuated total reflectance Fourier transform infrared spectroscopy (ATR FTIR) spectra were recorded using a FTIR spectrometer in the 4000–600 cm^−1^ range (NICOLET iS10, Thermo Scientific, USA) with deuterated triglycine sulfate (DTGS KBr) detector. A spectrum was recorded for each sample (PLCL, SP-PLCL) within the wave number region of 650–4000 cm^−1^, and 32 scans were collected with a resolution of 4 cm^−1^.

### Fabrication of coated ePTFE vascular graft

5% Hep-PLCL solution is prepared by dissolving Hep-PLCL in chloroform, and SP-PLCL is dissolved in chloroform to make 5% SP-PLCL solution. 5% Hep/SP-PLCL solution is prepared by dissolving the same amount of Hep-PLCL from previously made Hep-PLCL solution and the same amount of SP-PLCL from previously made SP-PLCL solution. The 1 cm cut ePTFE vascular grafts (6 mm in diameter, W.L. Gore & Associates, Inc.) are wetted with chloroform for 4 h with vortexing to coat better in inner (lumen side) and outer surface and pore, and then put into Hep-PLCL solution or SP-PLCL solution or Hep/SP-PLCL solution. After slightly vacuuming them for 10 seconds and vortexing them for 4 h, coated ePTFE vascular grafts are dried slightly to prevent the coating solution from flowing and put into excess methanol with stirring for 5 mins, and then dried in a vacuum oven for 3 days.

### Morphological analysis of coated ePTFE grafts

The surface morphology of polymer coated ePTFE vascular grafts was characterized by scanning electron microscopy (SEM, G-2 pro, Phenom). All samples were coated with gold prior to analysis and were observed in many random field. The average pore size was measured based on three SEM images at 450 times magnification for each group. The distance between fibril and fibril connecting transverse nodules was measured as pore size.

### Mechanical property of vascular grafts

Tensile strength of vascular grafts (ePTFE, Hep-PLCL coated ePTFE, SP-PLCL coated ePTFE, Hep/SP-PLCL coated ePTFE) (n = 3) were measured on a tensile testing machine (Instron 5988, USA) following ISO 7198 recommendation of vascular graft testing at a strain rate of 100 mm/min.

### *In vitro* cytotoxicity test

The cytotoxicity of biomolecules conjugated PLCL polymer was evaluated by WST-8 assay with Cell Counting Kit-8 (CCK-8) (Dojindo Laboratories Co. Ltd, USA) and cell (L929, ATCC CCL-1) proliferation and viability were assayed colorimetrically. WST-8 is reduced by dehydrogenases to give a yellow colored product in living cells. After samples were eluted in saline for 3 days, each extract solution was added to each well containing the same amount of cell in 48-well plate and then, it was incubated for 24 h at 37 °C in a 5% CO_2_ incubator. After removing the media, 280 µl of culture medium and 28 µl of the kit solution were added to each well and the samples were incubated for 4 h at 37 °C. The optical density of the resulting solution was measured at 450 nm by microplate ELISA reader (VERSA max, Molecular Devices; San Diego, USA).

### Endothelial cells adhesion and proliferation

Human umbilical vein endothelial cells (HUVECs; Lonza, Basel, Switzerland) (passage 6) (4 × 10^3^ cells/cm^2^) were seeded on coated surface (Hep-PLCL, SP-PLCL, Hep/SP-PLCL) and incubated for different times in incubator (37 °C, 5% CO_2_) and medium (EBM-2, Lonza) was replaced with fresh culture medium every 2–3 days. Samples were treated with the live/dead staining solution (2 µM calcein acetoxymethyl ester and 4 µM ethidium homodimer-1 in PBS) and incubated for 20 min at incubator. Live (green fluorescent) and dead (red fluorescent) cells images were obtained with a Zeiss LSM 700 confocal microscope (Carl Zeiss).

### Subcutaneous implantation of coated ePTFE vascular grafts

All animals were treated in accordance with standard operating protocols by the Institutional Animal Care and Use Committee at Biomedical Research Institute of Korea Institute of Science and Technology (KIST). All protocols for Animal Experiments were approved by the Institutional Review Board of Animal Experiments at Korea Institute of Science and Technology. All specimens were sterilized with ethylene oxide (EO) gas using a person EO35 system (Person medical, Gunpo-si, Gyeonggi-do, Korea).

Sprague-Dawley rats (balb/c, 6 week-old, male, 200–300 g; Nara Biotech) were anesthetized with ethyl ether. Dorsal skin of the rat was shaved with an electric clipper and disinfected with povidone iodine. The skin was vertically incised and muscle pockets were created on upper right and lower light and upper left and lower left side using surgical scissors without injury. After insertion of the teflon rod (5 mm diameter) inside of ePTFE graft to prevent grafts from folding, four types of grafts wetted in filtered 1X pbs for overnight (ePTFE, Hep-PLCL coated ePTFE, SP-PLCL coated ePTFE, Hep/SP-PLCL coated ePTFE) were implanted in the pockets randomly between skin and deep muscle and then, the incision was closed with a 3–0 black silk suture. After 2 weeks and 4 weeks, rats were sacrificed and grafts were explanted. Because the surrounding tissue could not be easily separated from the grafts, the vascular grafts were explanted with some surrounding tissue.

### MSCs recruitment

To investigate the MSCs recruiting activity of substance P and heparin coating, samples were stained with MSC markers (CD29, CD90). Samples were fixed with 10% buffered formalin, embedded in mixture of paraffin and EVA, and cross-sectioned into slices with 6 µm thickness for histological analysis. Samples were double stained with CD29 (goat, Integrin β1, Santa Cruz Biotechnology, Texas, USA, 1:100) and CD90 (mouse, Thy1, Abcam, Cambridge, United Kingdom, 1:200). Alexa Fluor 488 donkey anti-goat lgG (1:1000) and Alexa Fluor 594 rabbit anti-mouse (1:1000) were used for secondary anti

### Angiogenesis

To evaluate vascular cells density on coated ePTFE vascular grafts, endothelial cells (ECs) and smooth muscle cells (SMCs) were stained with von Willebrand factor antibody (rabbit, vWF, ab6994, 1:200) and α-smooth muscle actin antibody (mouse, α-SMA, ab7817, 1:200), respectively. Alexa Fluor 594 goat anti rabbit lgG (1:1000) and Alexa Fluor 488 goat anti-mouse (1:1000) were used for secondary antibodies. Also, samples were counterstained with DAPI to confirm the nuclei. After staining, the samples were observed in 5 random field at 200X magnification. The areas of positive stained samples were analyzed as the mean per unit area (µm²/ mm²) with the image J program (image processing software).

### Identification of M2 type macrophage

To identify M2 macrophage recruiting effect by coating, samples were double stained with CD68 (mouse, ab955, Abcam, 1:100) and CD206 (goat, sc-34577, Santa Cruz Biotechnology, 1:100). Alexa Fluor 594 rabbit anti mouse lgG (1:1000) and Alexa Fluor 488 donkey anti-goat (1:1000) were used for secondary antibodies. Also, samples were counterstained with DAPI to confirm the nuclei. After staining, the lumen and interior of ePTFE vascular grafts (samples) were observed in 5 random field at 200X magnification with a blinded rater in the border zone. The areas of positive stained samples were analyzed as the mean per unit area (µm²/ mm²) with the image J.

## Results

### Identification of covalent conjugation

**Figure 2:**
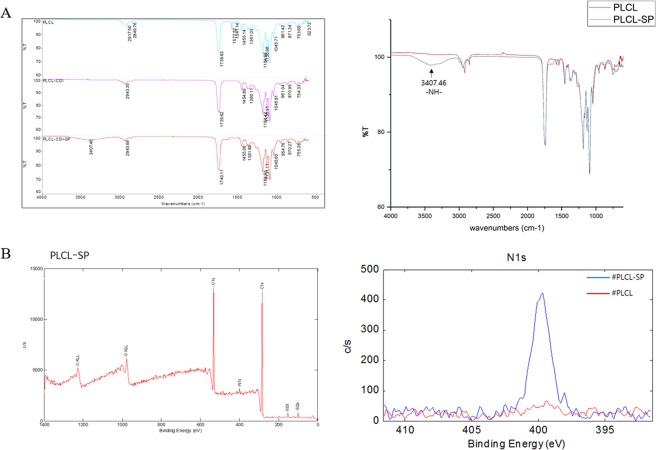
Confirmation of SP covalent conjugation with PLCL. (**A**) FTIR spectrum of PLCL, CDI-PLCL and SP-PLCL. The arrow indicated the main difference in absorb peaks and means primary amine (-NH-). (**B**) XPS spectra of SP-PLCL and high resolution spectra of the nitrogen peak (N1s).

The SP covalent conjugation with star-shaped PLCL was subjected to FT-IR and XPS analyses to understand the surface chemical composition (Fig. 2). The FT-IR spectra identified a new peak that does not exist in relation to the PLCL emergence at 3407 *cm*^−1^ in SP-PLCL, indicating that the primary amine (N-H stretching vibrations) of SP immobilized onto the terminal hydroxyl groups of the long PLCL polymer chain. Moreover, as determined by the XPS analysis, the intensity of the N1s peak increased in SP-PLCL compared to that in the PLCL, indicating that the nitrogen of SP conjugated to PLCL, which does not exist in PLCL. These data demonstrate that SP and PLCL are covalently well conjugated.

Hep-PLCL was synthesized by means of the covalent conjugation between the carboxyl group of heparin activated by DCC/DMAP and the terminal hydroxyl group of the long PLCL polymer chain. The covalent conjugation of heparin with PLCL was analyzed by a toluidine blue assay, and the heparin content on the surface of Hep-PLCL could be quantified through the standard curve of heparin. As a result, the heparin content on the surface of Hep-PLCL was found to be 0.16 µg/mg.

### Identification of ePTFE vascular graft coating

**Figure 3:**
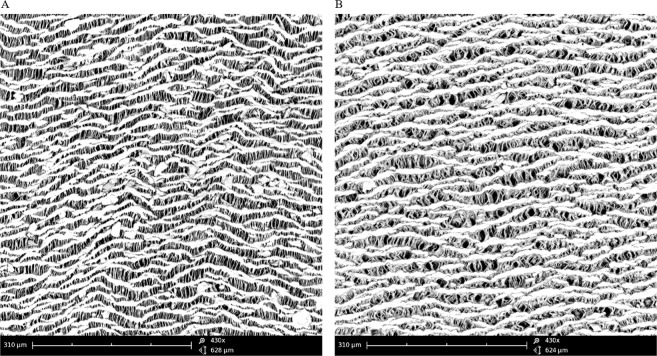
Surface morphology of teflon vascular graft. Non-coated ePTFE (**A**) is composed of thin Teflon threads, while coated ePTFE (**B**) is composed of a cluster of thin teflon threads. Scale bars: 100 µm (x450).

Scanning electron microscope (SEM) photomicrographs revealed the morphology of the vascular grafts. As shown in Fig. 3, the virgin ePTFE vascular graft is composed of transverse nodules connected by long fibrils. In contrast, in the coated ePTFE vascular graft, thin fibrils aggregated by polymer. Thus, the pore size increased because the distance between fibril and fibril was increased as mentioned above and the porosity decreased slightly, as the PLCL polymer acted as an adhesive. The average pore size of the ePTFE sample was 6.81 µm and the average pore size of the coated ePTFE was 12.8 µm. However, the mechanical properties of coated ePTFE vascular grafts were not significantly changed because the PLCL polymer was coated only lightly (Fig. S1).

**Figure 4:**
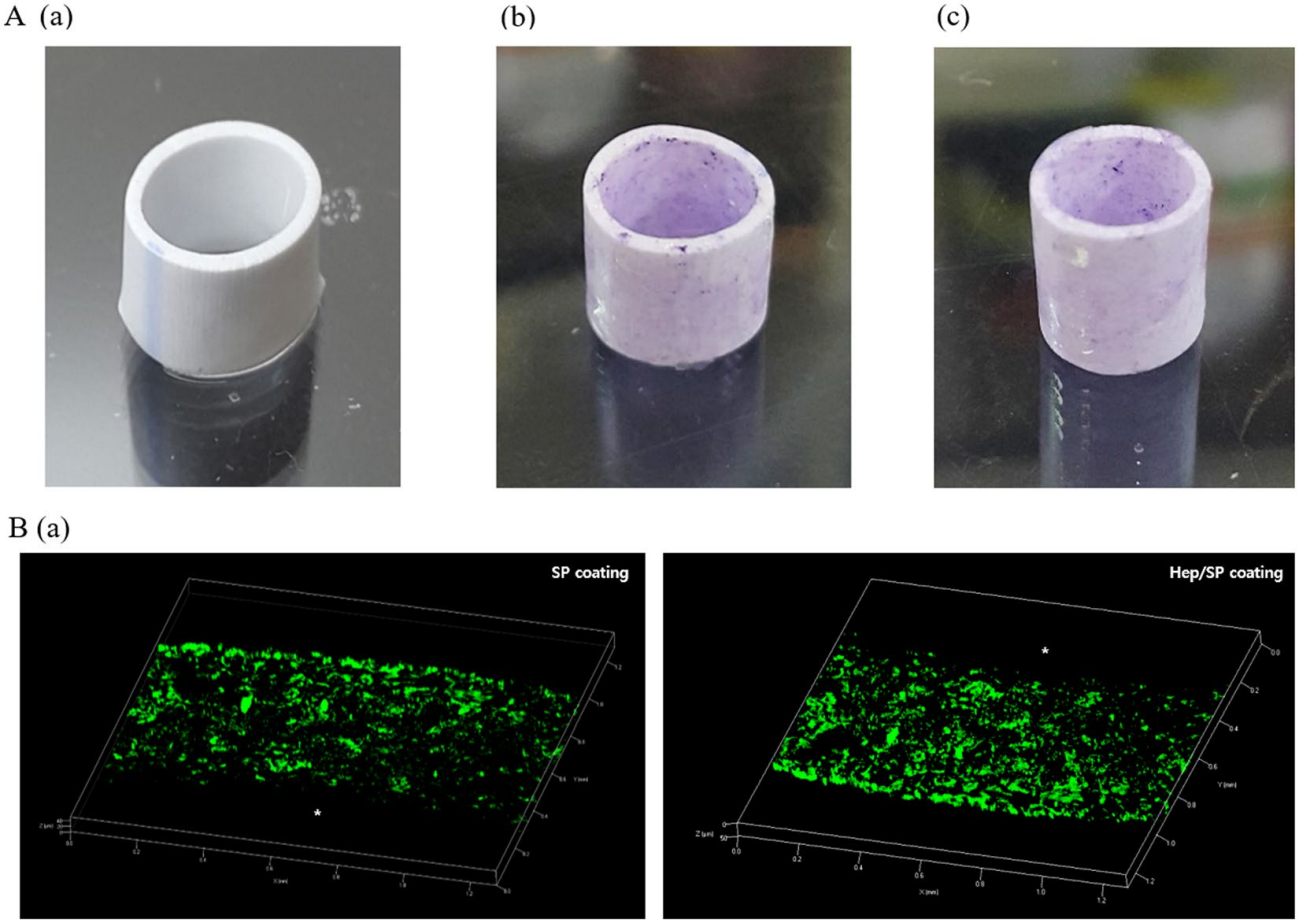
Confirmation of polymer (Hep-PLCL, SP-PLCL) coating on ePTFE vascular grafts by z-stack of confocal microscope. (**A**) Image of Teflon vascular graft stained with toluidine blue. Non coated ePTFE (A(a)) was not stained but, Hep-PLCL and Hep/SP-PLCL coated ePTFE (A(b) and A(c)) was stained with toluidine blue. (**B**) Immunostaining image of Teflon vascular graft stained with Anti-Substance P. SP was confirmed to be present in the inner pore and surface of the SP-PLCL vacuum-coated grafts. B(a) is SP-PLCL coated and B(b) is Hep/SP-PLCL coated ePTFE vascular grafts. *Represents the lumen of the scaffolds.

When the ePTFE and Hep-PLCL-coated ePTFE samples were stained with toluidine blue, the ePTFE did not turn purple due to the lack of heparin, whereas the Hep-PLCL- and Hep/SP-PLCL-coated ePTFE samples were stained purple uniformly, as shown in Fig. 4. Every coated ePTFE (Hep-PLCL and Hep/SP-PLCL) could be immersed in water because it was hydrophilized by heparin. As a result, it was confirmed that the coating was homogeneous on the ePTFE vascular grafts.

The SP-PLCL-coated ePTFE was immunostained with SP antibody to confirm the uniform coating of SP-PLCL on ePTFE artificial vascular grafts. A Z-stack of a confocal microscope was used to obtain a merged cross-section image with a thickness of 50 µm. As shown in Fig. 4, the SP-PLCL was uniformly coated inside the pores of the ePTFE artificial graft. As a result, the effect of substance P could be predicted in all parts of the ePTFE artificial vascular grafts, not only on the outer surface but also on the inner surface (lumen side) and on the pore surface.

### Histological analysis

**Figure 5:**
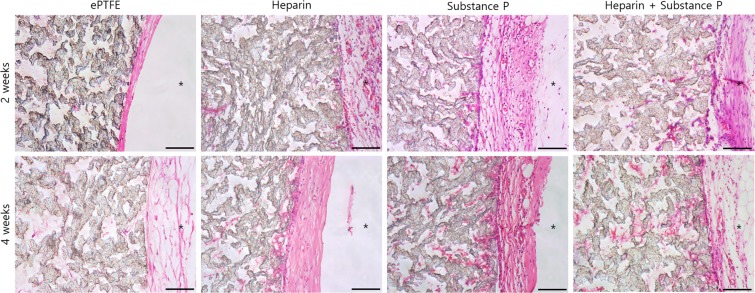
Hematoxylin and eosin staining for each group (non-coated ePTFE, Hep-PLCL coated ePTFE, SP-PLCL coated ePTFE, Hep/SP-PLCL coated ePTFE) after subcutaneous implantation for 2 weeks and 4 weeks). Coated ePTFE vascular grafts showed many recruited cells into the grafts and lumens. *Represents the lumen of the scaffolds. Scale bars: 100 µm (x200).

H&E staining of explanted grafts after two weeks and four weeks of implantation revealed that each coating enhanced the interaction with the cells (Fig. 5). Due to its bioinert nature, ePTFE did not interact with the host tissue and the cells scarcely infiltrated the pores of the ePTFE vascular grafts. In contrast, in the Hep-PLCL-coated ePTFE vascular grafts, a few cells infiltrated the pores of the grafts over time. However, in the SP-PLCL- and Hep/SP-PLCL-coated ePTFE vascular grafts, much more cells infiltrated the graft pores, and as time passed, more cells infiltrated the artificial vascular graft pores and proliferated.

### Recruiting of vascular cells through angiogenesis

**Figure 6:**
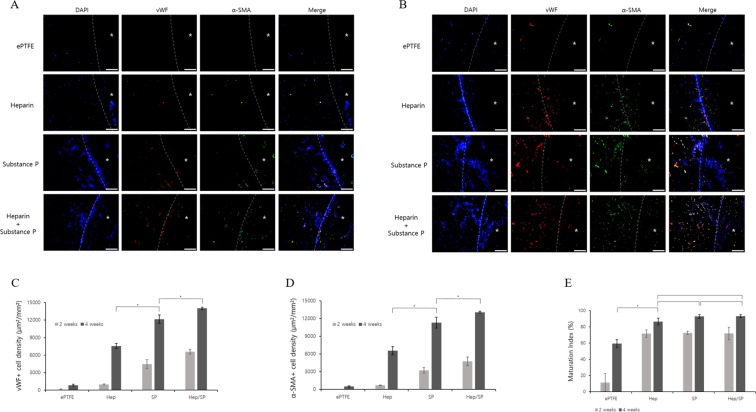
Effect of vascular cell recruiting by biomolecular coating on bio-inert ePTFE vascular graft. The expressed positive area of each groups means that remodeling of artificial vascular graft by ECs and SMCs. In immunofluorescence, ECs are stained with von Willebrand factor (vWF, red) and and SMCs are stained with α-Smooth muscle actin (α-SMA, green). (**A**) 2weeks and (**B**) 4weeks. Scale bars: 100 µm (x200). (**C**) Quantification of vWF+ cell density (µm²/mm²). (*p < 0.05) (**D**) Quantification of α-SMA+ Qcell density (µm²/mm²). (*p < 0.05) (**E**) Quantification of the maturation index was based on comparison of the percentage of α-SMA+ vessels to the total number of vessels. (*p < 0.05). *Represents the lumen of the scaffolds.

The vascular cell (ECs, SMCs) recruiting effect of the biomolecular coating of the ePTFE artificial vascular grafts was evaluated by the double staining of vWF and α-SMA (Fig. 6). The vWF+ cell density (µm^2^/mm^2^), α-SM+ elcell density (µm^2^/mm^2^), and maturation index (%) values of each group were quantified at two weeks and four weeks after implantation. In the ePTFE vascular grafts (non-coated ePTFE), vascular cells scarcely grew, whereas in the other groups, more vascular cells grew over time. At two weeks, the vWF+ cell density of Hep/SP-PLCL-coated ePTFE was 6.86 times that of the Hep-PLCL-coated ePTFE and 1.48 times that of the SP-PLCL-coated ePTFE (6589.40 ± 424.93 µm²/mm² in the Hep/SP-PLCL group, 4463.82 ± 742.51 µm²/mm² in the SP-PLCL group, 960.83 ± 86.88 µm²/mm² in the Hep-PLCL group, and 81.26 ± 69.89 µm²/mm² in ePTFE). In addition, at four weeks, the vWF+ cell density of the Hep/SP-PLCL-coated ePTFE was 1.86 times that of the Hep-PLCL-coated ePTFE and 1.15 times that of the SP-PLCL-coated ePTFE (14028.69 ± 192.70 µm²/mm² in the Hep/SP-PLCL group, 12175.74 ± 718.05 µm²/mm² in the SP-PLCL group, 7560.64 ± 441.15 µm²/mm² in the Hep-PLCL group, and 830.50 ± 163.32 µm²/mm² in ePTFE). Thus, Hep/SP-PLCL coating was most effective in recruiting of endothelial cells (ECs) compared to other groups.

At two weeks, the α-SMA+ cell density of the Hep/SP-PLCL-coated ePTFE was 6.92 times that of the Hep-PLCL-coated ePTFE and 1.47 times that of the SP-PLCL\-coated ePTFE (4757.09 ± 757.43 µm²/mm² in the Hep/SP-PLCL group, 3228.04 ± 452.86 µm²/mm² in the SP-PLCL group, 687.90 ± 37.56 µm²/mm² in the Hep-PLCL group, and 5.21 ± 2.89 µm²/mm² in ePTFE), while at four weeks, the α-SMA+ cell density of the Hep/SP-PLCL-coated ePTFE was 1.99 times that of the Hep-PLCL-coated ePTFE and 1.16 times that of the SP-PLCL-coated ePTFE (13063.44 ± 155.77 µm²/mm² in the Hep/SP-PLCL group, 11308.50 ± 914.75 µm²/mm² in the SP-PLCL group, 6557.69 ± 684.18 µm²/mm² in the Hep-PLCL group, and 495.94 ± 114.51 µm²/mm² in ePTFE). Similarly, Hep/SP-PLCL coating was most effective in smooth muscle cells (SMCs) recruiting.

The maturation index of each group was calculated to compare the maturation of the vessels based on the double staining results at two weeks and four weeks. At two weeks, the maturation index is 71.92% for the Hep/SP-PLCL-coated ePTFE, 72.54% for the SP-PLCL-coated ePTFE, and 71.83% for the Hep-PLCL-coated ePTFE, but there were no differences between the groups. Moreover, at four weeks the values are 93.13% for the Hep/SP-PLCL-coated ePTFE, 92.80% for SP-PLCL-coated ePTFE, and 86.59% for the Hep-PLCL-coated ePTFE. Based on these results, the Hep/SP-PLCL coating is most effective for vascular cell recruiting through angiogenesis.

### Stem cell recruiting ability of the coating

**Figure 7:**
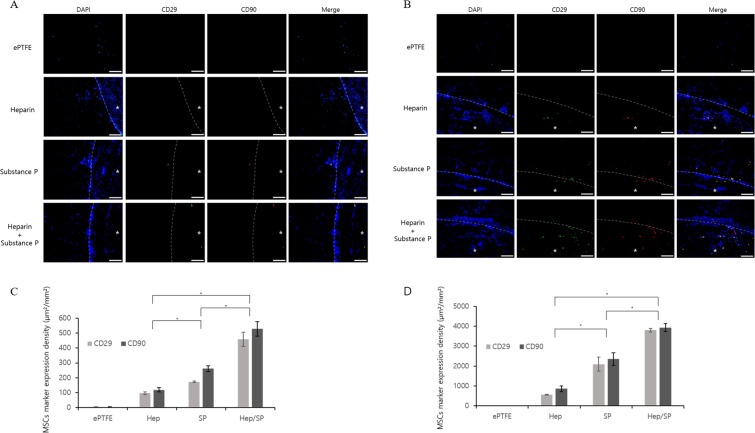
Mesenchymal stem cells (MSCs) recruitment of bioactive molecular coating. SP-PLCL and/or Hep-PLCL coating is effective for the recruitment of MSCs into bio-inert artificial vascular grafts. Representative images of artificial vascular grafts from each group after CD29, CD90 staining. (**A**) 2weeks and (**B**) 4weeks after implantation. Scale bars: 100 µm (x200). (**C**) Quantification of MSCs expression area of each group at 2 weeks (µm²/mm²). (**D**) Quantification of MSCs expression area of each group at 4 weeks. (*p < 0.05). *Represents the lumen of the scaffolds.

We used double staining for the MSC specific markers CD29 and CD90 to identify the stem cell recruiting capabilities of the Hep-PLCL, SP-PLCL and Hep/SP-PLCL coatings. A quantitative analysis showed that there were few MSCs at two weeks, whereas many more MSCs were recruited at four weeks (Fig. 7). The ePTFE group showed few double-stained cells inside the graft and lumen areas. In the Hep-PLCL coating and SP-PLCL coating groups at four weeks, some double-stained cells were observed; whereas the SP-PLCL coating group showed more double-stained cells than the Hep-PLCL coating group. The Hep/SP-PLCL coating group showed the highest quantity levels of double-stained cells (MSCs) in all of the groups. Therefore, the Hep-PLCL coating was found to be some effective but, the SP-PLCL coating were found to be very effective for recruiting MSCs, with the Hep/SP-PLCL coating most effective.

### Induction of regeneration by the recruiting of M2 macrophages

**Figure 8:**
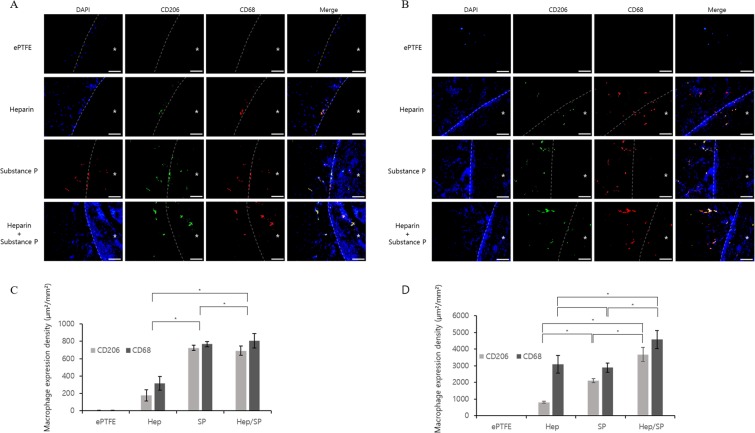
Fluorescence microscopy images for conformation of M2 macrophages recruitment by bioactive molecular coating. Double stained images of CD68 and CD206 mean M2 macrophage. (**A**) 2weeks and (**B**) 4weeks after implantation. Scale bars: 100 µm (x200). (**C**) Quantification of macrophage expression density of each group at 2 weeks (µm²/mm²). (**D**) Quantification of MSCs expression density of each group at 4 weeks. (*p < 0.05). *Represents the lumen of the scaffolds.

To confirm the suppressed pro-inflammatory effects and increased anti-inflammatory effects caused by the coatings, we undertook double staining for CD206 and CD68 (Fig. 8). The non-coated ePTFE is negative for CD68 and CD206 with few cells because it is bio-inert, and the Hep-PLCL coating group has many CD68+/CD206− cells (M1 macrophages) as compared to the number of CD68+/CD206+ cells (M2 macrophages). In contrast, the SP-PLCL coating group and the Hep/SP-PLCL coating group have many CD68+/CD206+ cells. At two weeks, the Hep/SP-PLCL coating group and the SP-PLCL coating group have more M2 phenotypic macrophages and fewer CD68+/CD206− cells than the Hep-PLCL coating. At four weeks, Hep/SP-PLCL coating and SP-PLCL coating led to much more M2 phenotypic macrophages compared to the outcome at two weeks, while the Hep/SP-PLCL coating had the most M2 phenotypic macrophages. Hence, we can confirm that the Hep/SP-PLCL coating and the SP-PLCL coating cause monocytes to differentiate more into M2 macrophages. These M2 macrophages have reparative/anti- inflammatory effects and stimulate cell migration and modulate cell proliferation and differentiation and induce angiogenesis^34^.

## Discussion

The formation of an endothelial layer in the lumen is crucial to enhance long-term patency, and it is also important when attempting to exploit vascular grafts with small dimeters. Various studies developed artificial vascular grafts, such as TEVG and cell free vascular grafts. However, the results of these studies are not yet ready to apply in clinical practice owing to many unresolved problems; the development of these grafts is urgent, but it will take a very long time. Thus, in order to save numerous lives of people who are suffering now, we must initially overcome the limitations of artificial vascular grafts made of ePTFE, which are now the most commonly used type in clinical practice. Therefore, through coatings, we attempted to overcome the limitations of the poor long-term patency caused by the super-hydrophobicity of ePTFE.

As results of XPS, FT-IR, SEM and staining analysis, heparin and substance P were effectively conjugated with PLCL, and these polymers were lightly coated onto the pore surface as well as the inner (lumen side) and outer surfaces of the ePTFE vascular grafts by a vacuum coating method. Consequently, the porous scaffold of the coated ePTFE provided sufficient space in which cells can infiltrate and grow through recruitment and migration, as well as space for the deposition of vascular ECM. Large pore size of coated ePTFE vascular grafts facilitate infiltration of capillaries from the surrounding tissue by angiogenesis, endothelialization and smooth muscle cell formation (Fig. 6). But, small pore size of non-coated ePTFE vascular grafts may not allow the cell penetration after implantation (Fig. 5). Numerous studies insist that large pore size (10~30 µm) provide superior graft healing, stability and patency compared to small pore size^35–37^. Tara *et al*. proved large pore grafts (30 µm) showed a well-organized neointima after 12 months, and no calcification in contrast with small pore grafts (5 µm)^38^. Thus, we anticipate that pores (about 13 µm) of coated ePTFE graft are not enough to cause bleeding and provide effective luminal endothelialization and smooth muscle cells infiltration.

Because the coated samples were implanted subcutaneously, the outer surfaces of the ePTFE vascular grafts were in contact with the subcutaneous tissue, causing the surrounding tissue migration to be maximized. However, because the focus of research is to clarify only the recruiting effect of the cells such as vascular cells, stem cells and immune cells by coating, not the formation of an endothelial layer in lumen of grafts due to subcutaneously implantation, we observed the lumens and insides of the ePTFE vascular grafts. When the coated vascular grafts were implanted subcutaneously, a Teflon rod was inserted inside of the vascular grafts to prevent the vascular grafts from being pressed, which made it difficult slightly for the cells to infiltrate into the lumen, unlike the outer surface, which is in contact with the subcutaneous tissue. But, when the bioactive molecule of the coated vascular grafts solely recruited cells effectively, could the cells infiltrate and grow into the lumen (between grafts and teflon rod) and vascular grafts. Therefore, the cells present in the lumen and the cells inside the vascular grafts are cells recruited by substance P and heparin. With H&E staining (Fig. 5), most cells infiltrated into the vascular grafts in the Hep/SP-PLCL coating group and many cells infiltrated in the SP-PLCL coating group, with some cells infiltrating in the Hep-PLCL coating group at four weeks, unlike in the non-coated group. From these results, we confirmed that the coatings created a microenvironment in which bioinert artificial vascular grafts can interact with the body. These interactions between the host and grafts induced regeneration, likely leading to the remodeling of the ePTFE vascular grafts. In the current study, because we implanted subcutaneously, so many cells are recruited in lumen of grafts, but *in-situ* implantation into the blood vessels in later experiments is expected to exhibit a different pattern because of blood flow.

As mentioned above, in order to form an endothelial layer in the lumen, endothelial cells and smooth muscle cells must grow together in ePTFE vascular grafts like native vessels so that they are not washed out by the blood flow. Hong *et al*. demonstrated that EC retention on ePTFE grafts in the blood flow is significantly better when ECs are seeded on top of SMCs than when EC are seeded alone, as SMCs adhere well to the graft wall, unlike ECs, and ECM secreted by SMCs enhance cell attachment through interactions between membrane proteins such as integrin and the ECM^8^. It is reported that PLCL is highly compatible with the proliferation of ECs and SMCs^10,39^. Therefore, we created an environment in which ECs and SMCs could stably attach to the ePTFE vascular grafts and grow together through biomolecular conjugated PLCL.

In order to demonstrate that the coatings can create an environment in which ECs and SMCs grow together and recruit both ECs and SMCs, we undertook double staining with vWF and α-SMA (Fig. 6). A double coating of SP-PLCL and heparin-PLCL was most effective for recruiting ECs and SMCs, a coating of only SP-PLCL was also very effective, and a heparin-PLCL coating was quite effective because substance P stimulates the synthesis and release of angiogenic cytokines in monocytes and heparin has biological activities associated with its interaction with cytokines and growth factors^22,40^. Kohara *et al*. demonstrated that the controlled release of substance P induces angiogenesis through the recruitment and activation of circulating cells with angiogenic activities^17^. Kim *et al*. proved that a substance P treatment induces the formation of blood vessels through the enhanced recruitment of MSCs, EPC, pericytes and HSCs^12^. Wiedermann *et al*. demonstrated that substance P stimulates neo-vessel formation through the induction of endothelial cell proliferation and differentiation^41,42^. Heparin was coated to prevent early-stage thrombosis, which is the most serious problem associated with transplantation in actual blood vessels, by inhibiting protein adsorption and platelet adhesion. However, because heparin interacts with various cytokines and growth factors through specific interactions, as noted above, it was also found to be effective during the recruitment of vascular cells such as ECs and SMCs in two heparin-coated groups^20,24–29^. In this study, vascular cells recruited into ePTFE vascular grafts did not derive from the migration of cells in the anastomosed vessels of *in situ* implants but derived instead from neo-vessels newly produced by the coating. Based on this result, it can be predicted that in upcoming experiments, when the sample is *in-situ* implanted into the blood vessel, the effect of recruiting the endothelial cells and smooth muscle cells into the ePTFE vascular grafts will be maximized. Thus, it is expected that an endothelial layer will be formed in the lumen of the *in-situ* implanted artificial vascular grafts.

There were no MSCs in the non-coating group (ePTFE) and only a few MSCs in the Hep-PLCL coating group, whereas many MSCs were recruited in the Hep/SP-PLCL coating group and in the SP-PLCL coating group (Fig. 7). These results prove that the substance P effectively causes the recruiting of MSCs. Hong *et al*. demonstrate that substance P stimulates stem cells to move into the blood stream and induces the recruiting and proliferation of MSCs to injured sites for wound healing^43,44^. Our results demonstrated that substance P is released by breaking the covalent bond of SP-PLCL, after which it recruits MSCs into the artificial vascular grafts. In the Hep/SP-PLCL coating group and the SP-PLCL coating group, there were only a few MSCs at two weeks, but over time, there were many more MSCs at four weeks. This result occurs because the stem cell homing effect was enhanced gradually by the sustained release of substance P due to the covalent conjugation of substance P and PLCL^11^. Another reason for the recruiting of significantly more MSCs at four weeks compared to two weeks is the angiogenesis effect, which is much better at four weeks than at two weeks, as shown in Fig. 6. Thus, many MSCs could be recruited inside of the grafts through these newly created neo-vessels into the grafts^44^. Many studies have demonstrated that host stem cells promote tissue repair by differentiating into multiple lineages and also by the secreting of factors that enhance regeneration, stimulate proliferation and differentiation, and decrease inflammation and immune reactions^45–47^. MSCs have the potential to modulate macrophage polarization from the inflammatory M1 phenotype toward the anti-inflammatory and tissue-regenerative M2 phenotypes by secreting various factors, such as IL-10, transforming growth factor β1 (TGF-β1), indoleamine-2,3-dioxygenase (IDO) and prostaglandin E2 (PGE2). They also have the potential to reduce apoptosis^48–50^. Moreover, it was proved that MSCs promote angiogenesis through the production of many angiogenic factors; they stimulate the modulation of endothelial cell migration and induce vessel remodeling as well as capillary proliferation^45,51^. Thus, we expected that MSCs recruited into vascular grafts by the coating would enhance the remodeling of the artificial graft by regulating immunity, inducing angiogenesis and differentiating into vascular cells.

Lim *et al*. demonstrate that substance P serves as a novel cytokine because it causes pro-inflammatory macrophages (M1) to differentiate into tissue-repairing macrophages (M2)^52^. M2 macrophages induce anti-inflammatory and regenerative responses by regulating IL-10, TGF-β, Arg-1, MMP13, and macrophages and contribute to the resolution of inflammation, tissue remodeling and angiogenesis, while M1 macrophage play a pro-inflammatory role through the production of pro-inflammatory cytokines such as IL-6, TNF-α, and nitric oxide^11,53–55^. It has been reported that macrophages are a source of mitogens and angiogenic factors and that they proceed ahead of sprouting vessels and form tunnels to guide neovascularization *in vivo*^56–59^. It was proved that the coating inhibited inflammation and promoted regeneration in results that also showed that SP-PLCL and Hep/SP-PLCL coatings inhibited the infiltration of M1 macrophages and enhanced the recruitment of M2 macrophages (Fig. 8). In addition, it can be expected that macrophages recruited into vascular grafts will stimulate the vascularization of the artificial vascular grafts and eventually undergo remodeling as functional vessels through the secretion of angiogenic factors such as bFGF, TGF-β, and VEGF^60,61^. Intima hyperplasia is caused by continued inflammatory response^62^. But, because the Hep/SP-PLCL coating suppresses the pro-inflammatory response, it can inhibit intimal hyperplasia caused by activation of SMCs by inflammation. Also, Intimal hyperplasia can be suppressed because endothelium formed by Hep/SP coating inhibits thrombus formation and inflammation, and reduce SMC proliferation and migration and because ECs maintain SMCs in a quiescent state by secreting NO, TGF-β^63–66^.

In summary, conventional ePTFE artificial vascular grafts made of hydrophobic teflon inhibited early thrombosis, but after a long time, thrombosis developed and the blood vessels became clogged again. This poor long-term patency arises because vascular cells do not grow due to the lack of interaction with the body, unlike actual blood vessels. Therefore, we caused ePTFE vascular grafts to interact with the body through the use of a coating of Hep/SP-PLCL. Hence, we caused bioinert ePTFE artificial vascular grafts to be remodeled into functional vessels. The Hep/SP-PLCL coating recruited endogenous stem cells and progenitor cells into the blood vessels and stimulated the angiogenesis and migration of peripheral vascular cells. Therefore, ePTFE vascular grafts were composed of vascular cells inside the grafts, akin to native vessels. MSCs recruited by the coating inhibited polarization to pro-inflammatory cells and promoted the polarization of anti-inflammatory cells, leading to tissue repair. Thus, Hep/SP-PLCL-coated ePTFE vascular grafts are likely to be remodeled into functional artificial vessels such as native vessels with good long-term patency when it is implanted *in-situ*.

This coating technique can be applied to small-diameter ePTFE vascular grafts, which are urgently needed clinically but which are also not usable due to thrombosis and occlusion caused by the inability of vascular cells to grow. Hep/SP-PLCL coatings inhibit early thrombosis by heparin^20–23^ and allow ePTFE vascular grafts to interact with the body, resulting in the regeneration of smooth muscle cells and an endothelial layer thus to overcome the limitations of small-diameter ePTFE vascular grafts.

Additionally, these coatings can be applied to all implantable medical devices to help overcome the limitations caused by poor performance after implantation. The foreign body response can be overcome because the Hep/SP-PLCL coating prevents the adsorption of proteins at the interface between the host and device while also regulating immunity and inducing the formation of autologous tissue. In upcoming research, we plan to validate the effect of our coating on other medical devices.

## Conclusion

Modulating inflammation and inducing angiogenesis and MSC recruitment are key therapeutic strategies in regenerative medicine. It was demonstrated here that Hep/SP-PLCL coatings transform bioinert artificial vascular grafts into functional blood vessels akin to native vessels through graft remodeling by interacting with the body. These coatings form a virtuous cycle by which angiogenesis is induced in the grafts, MSCs are recruited through neo-vessels, MSCs induce polarization to M2 macrophages and M2 macrophages stimulate angiogenesis. These finding suggests the possibility of artificial medical devices recognized as autologous tissue in the body through the induction of autologous tissue formation on device surfaces with regulation of the immune response.

## Supplementary information

Supplementary data
