# Supplementary material for: The effect of Substance P/Heparin conjugated PLCL polymer coating of bioinert ePTFE vascular grafts on the recruitment of both ECs and SMCs for accelerated regeneration
Source: Sci Rep. 2019 Nov 19;9:17083. doi: 10.1038/s41598-019-53514-6 (PMC6863833; doi:10.1038/s41598-019-53514-6)
Supplement: Supplementary file 1 — Supplementary data [file 41598_2019_53514_MOESM1_ESM.docx]

**Supplementary Data**

Title of the article: The effect of Substance P/Heparin conjugated PLCL polymer coating of bioinert ePTFE vascular grafts on the recruitment of both ECs and SMCs for accelerated regeneration

**Names of authors**

Donghak Kim^1,2^, Justin Jihong Chung^2^, Youngmee Jung^2,3^, Soo Hyun Kim^1,2,3,*^

* : Corresponding author

**Addresses of Establishments**

^1^KU-KIST Graduate School of Converging Science and Technology, Korea University, 145 Anam-ro, Seongbuk-gu, Seoul, 02841, Republic of Korea

^2^Center for Biomaterials, Korea Institute of Science and Technology, Seoul 02792, Republic of Korea

^3^Department of Biomedical Engineering, Korea University of Science and Technology (UST), Daejeon 305-350, Republic of Korea


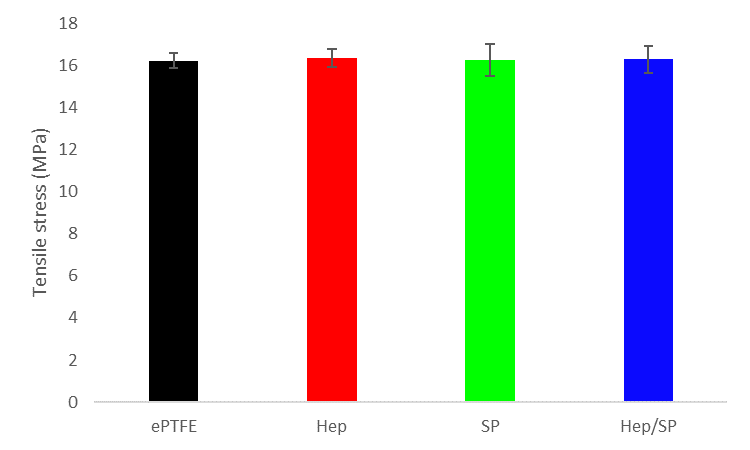


Fig. S1. The tensile strength of vascular grafts (ePTFE, Hep-PLCL coated ePTFE, SP-PLCL coated ePTFE, Hep/SP-PLCL coated ePTFE).

Tensile strength of vascular grafts (ePTFE, Hep-PLCL coated ePTFE, SP-PLCL coated ePTFE, Hep/SP-PLCL coated ePTFE) were measured by using a tensile testing machine (Instron 5988, USA) following ISO 7198 recommendation of vascular graft testing at a strain rate of 100mm/min. As shown as Fig. S1, the tensile stress of coated ePTFE vascular grafts is not significantly different from that of non-coated ePTFE. Therefore, the properties of coated ePTFE vascular grafts were not significantly changed because the PLCL polymer was coated lightly.


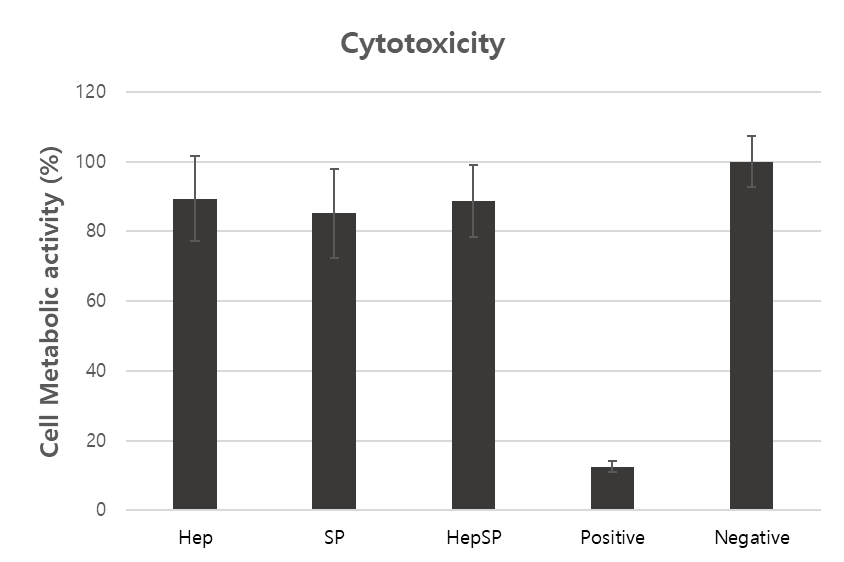


Fig. S2. Cytotoxicity test with WST assay (Hep-PLCL coated ePTFE, SP-PLCL coated ePTFE, Hep/SP-PLCL coated ePTFE, ZDEC polyurethane film extract solution, saline solution).

The cytotoxicity assay was done with the WST assay. Compared to negative control (saline solution), WST-8 formazan absorbance has distinctly decreased in positive control (ZDEC polyurethane film). But, the cell viability of every polymer coated groups (Hep-PLCL, SP-PLCL, Hep/SP-PLCL) remained close to that of negative control group. Thus, it was observed that every biomolecule conjugated polymer coating barely showed cytotoxicity against L929 (mouse fibroblast). These results demonstrate that the biomolecule conjugated PLCL polymer coating on ePTFE has good biocompatibility.

**A B**


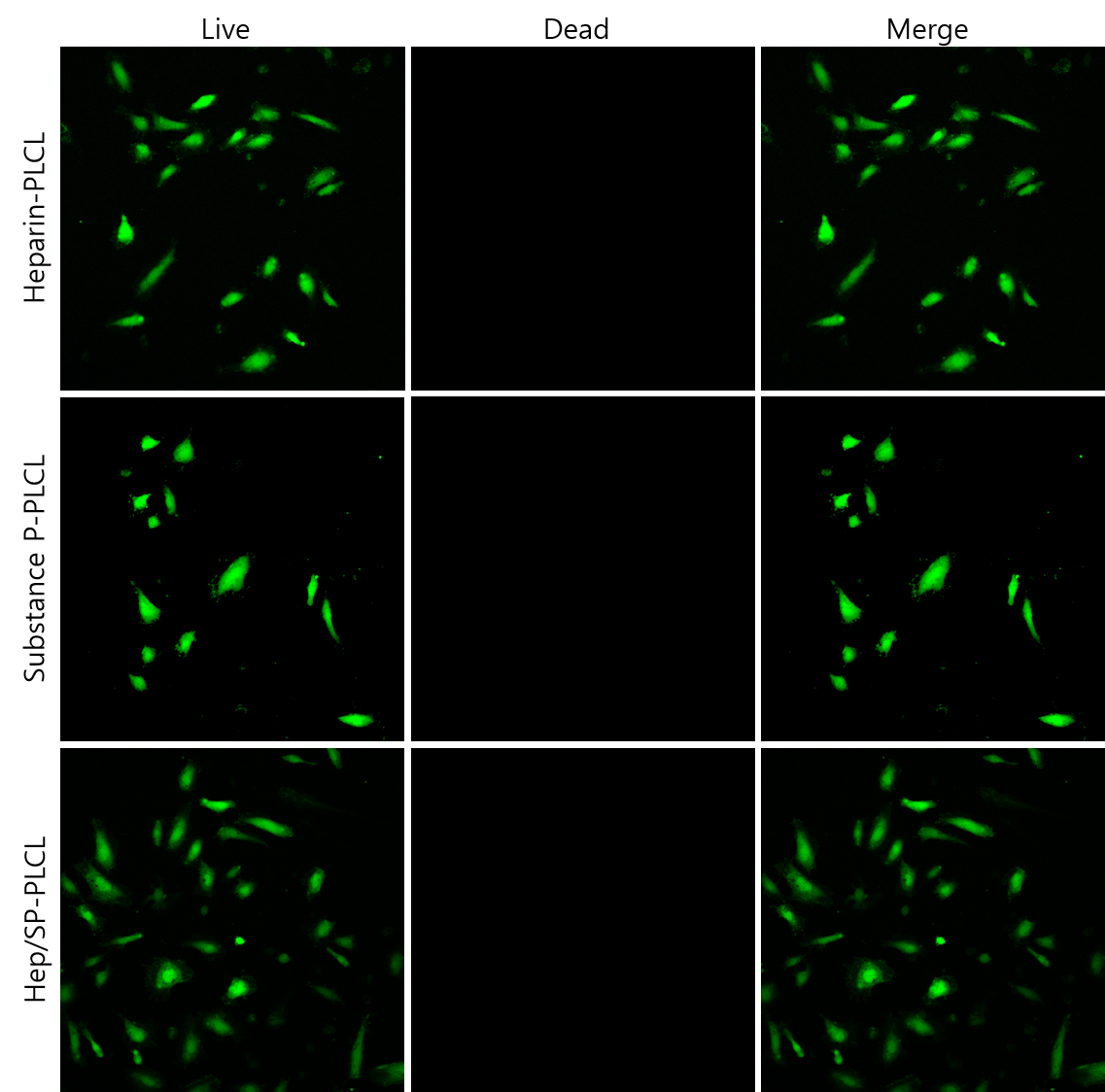

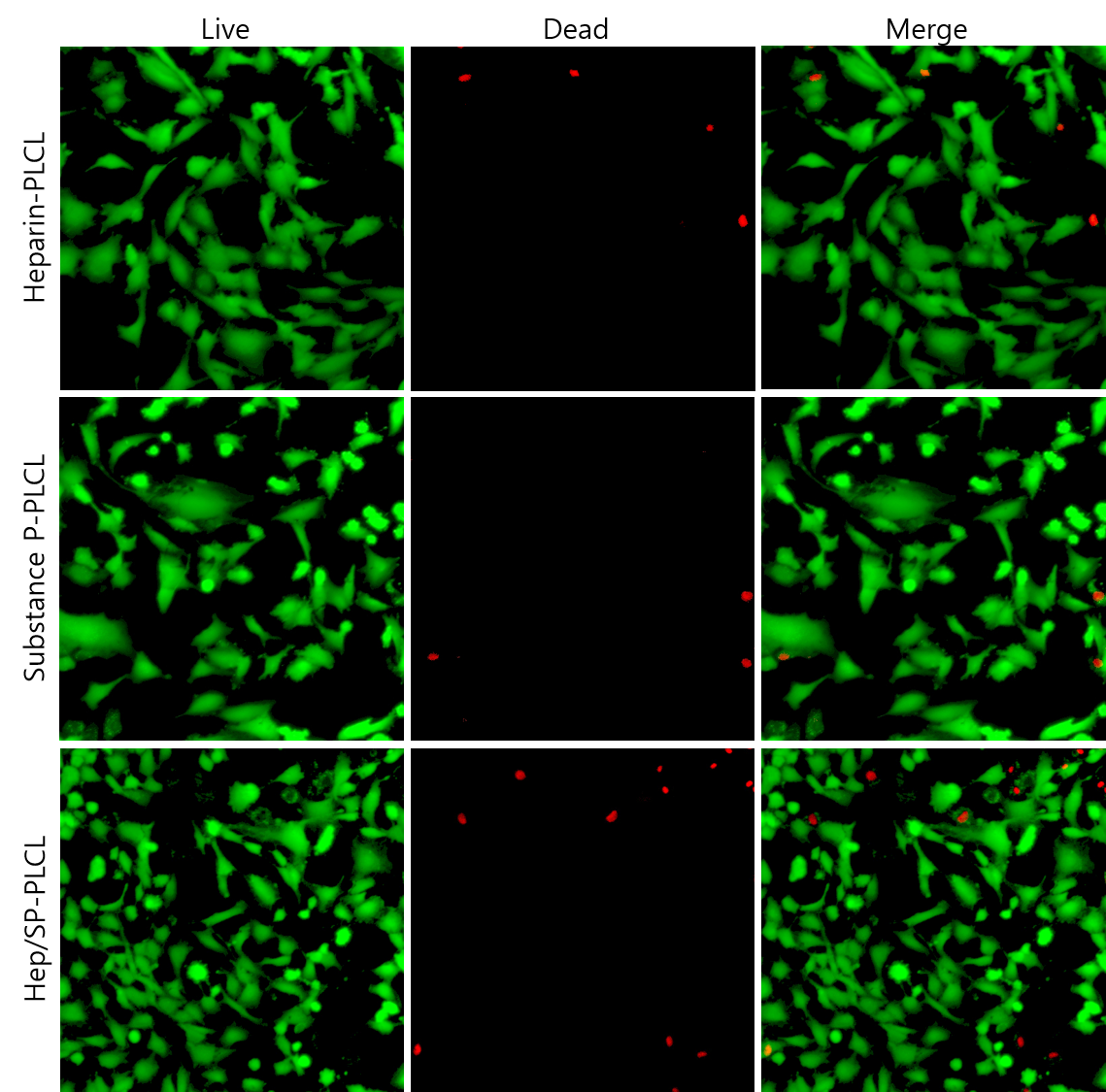


Fig. S3. Live/Dead assay images of HUVECs seeded on the coated surface. (A) 1day and (B) 7 days after seeding.

To investigate the regeneration effect of coating, HUVECs were seeded on the coated surface. If HUVECs can adhere stably on coated vascular grafts in situ, proliferate and endothelialize rapidly, grafts will be regenerated into functional vessels because they control protein adhesion, platelet activation and thrombosis. The performed in vitro cell assays showed that all coated surfaces (Heparin-PLCL, SP-PLCL, and Hep/SP-PLCL group) were effective for endothelial cells adhesion and proliferation (Fig. S3.). The Hep-PLCL coated group displayed higher HUVECs adhesion in vitro than SP-PLCL coated group because heparin has integrin and selectin on cell membrane interact with heparin [1]. In particular, a double coating of SP-PLCL and heparin-PLCL (Hep/SP-PLCL) was most effective for adhesion of endothelial cells. The more preferable adhesion of HUVECs on Hep/SP-PLCL coated group can be attributed to the presence of more functional groups such as COO^-^, SO_3_^-^, NH_3_^+^ at the coated surface. After 7days of seeding, the HUVECs proliferated quickly and eventually formed a whole endothelial cell layer on the all coated surface. Thus, these results mean that coating can remodel the surface to effectively interact with endothelial cells and can regenerate the vascular grafts in vivo.

1 Choi, D. H. *et al.* Growth factors-loaded stents modified with hyaluronic acid and heparin for induction of rapid and tight re-endothelialization. *Colloids Surf B Biointerfaces* **141**, 602-610, doi:10.1016/j.colsurfb.2016.01.028 (2016).
